# Low literacy skills in adults can be largely explained by basic linguistic and domain-general predictors

**DOI:** 10.3389/fpsyg.2024.1422896

**Published:** 2024-09-04

**Authors:** Réka Vágvölgyi, Moritz Sahlender, Hannes Schröter, Benjamin Nagengast, Thomas Dresler, Josef Schrader, Hans-Christoph Nuerk

**Affiliations:** ^1^Cognitive and Developmental Psychology Unit, Centre for Cognitive Science, RPTU Kaiserslautern-Landau, Kaiserslautern, Germany; ^2^German Institute for Adult Education - Leibniz Centre for Lifelong Learning (Deutsches Institut für Erwachsenenbildung - Leibniz-Zentrum für Lebenslanges Lernen e.V.), Bonn, Germany; ^3^Adult Cognition and Learning, Faculty of Psychology, FernUniversität in Hagen, Hagen, Germany; ^4^Hector Research Institute of Education Sciences and Psychology, University of Tuebingen, Tuebingen, Germany; ^5^LEAD Graduate School & Research Network, University of Tuebingen, Tuebingen, Germany; ^6^Department of Psychiatry and Psychotherapy, University of Tuebingen, Tuebingen, Germany; ^7^German Center for Mental Health (DZPG), Partner Site Tuebingen, Tuebingen, Germany; ^8^Department of Education, University of Tuebingen, Tuebingen, Germany; ^9^Department of Psychology, University of Tuebingen, Tuebingen, Germany

**Keywords:** functional illiteracy, low literacy, poor reading adults, struggling adult readers, text comprehension, domain-general functions, numeracy, transparent orthography

## Abstract

**Introduction:**

Despite having sufficient formal education, a large group of people cannot complete everyday tasks like reading, writing, or making basic calculations. Regarding reading, millions of people are not able to understand more complex texts despite the ability to read simple words or sentences; they have low literacy skills. Even though this problem has been known for decades, the causes and predictors of their poor reading comprehension skills are not fully explored. Socioeconomic, sociodemographic, and reading-related (i.e., linguistic) factors, especially of English-speaking participants and thus users of an opaque orthography, were often assessed. The goal of this study was to examine which linguistic, domain-general, or numerical factors predict substandard complex text reading as the core symptom of low literacy skills in adulthood.

**Methods:**

To this end, we assessed a group of German-speaking participants—users of a transparent orthography—who are at risk for complex text reading deficits.

**Results:**

The results indicated that linguistic variables (reduced word/pseudoword reading, weaker oral semantic and grammatical comprehension), working memory, and age predicted lower performance in text comprehension. This model explained 73% of the total variance, indicating that most of the deficits in complex text reading can be explained by a group of basic underlying linguistic and domain-general factors.

**Discussion:**

We conclude that interventions for adults with low literacy skills and others at risk for complex text reading deficits should address word/pseudoword reading and focus on both written and oral comprehension.

## 1 Introduction

Basic literacy competences, such as reading, writing, and calculation, are essential for participation in a modern society. Although primary and secondary education is accessible and obligatory in developed countries, even the welfare states in Europe have a considerable percentage of adults struggling with literacy skills (e.g., Finland: 10.6%, England: 16.4%, Germany: 12.1–17.5%; Grotlüschen and Buddeberg, 2020; OECD, 2019b).

The current study focuses on a subgroup previously referred to as functional illiterates (e.g., Boltzmann and Rüsseler, 2013; Grotlüschen and Buddeberg, 2020; for a review see Vágvölgyi et al., 2016). However, as the term is considered stigmatizing (Grotlüschen and Buddeberg, 2020) and because the naming and the operationalization of functional illiteracy varies considerably (Perry et al., 2017; Rosen, 2022), the more descriptive term “adults with low literacy skills” will be used throughout the manuscript. We define them here as individuals who have undergone at least some years of education in which they should have acquired basic literacy skills but still struggle with written materials as adults, especially those requiring reading comprehension skills (Bulajić et al., 2019; Vágvölgyi et al., 2016).

### 1.1 Underlying components of poor reading comprehension

For an efficient comprehension of texts more than single-word recognition is required; it also assumes the ability to comprehend complex language in any form. If someone can read well but is generally unable to understand more complex narratives in other inputs, this person would also have problems with reading complex texts (for the theoretical model, see Simple View of Reading; Gough and Tunmer, 1986).

Socioeconomic and sociodemographic factors have been shown to affect literacy acquisition in childhood (e.g., Hemmerechts et al., 2016) as well as literacy proficiency in adulthood (e.g., OECD, 2019b). The German LEO 2018 study found age (older than 45 years), gender (male), language (non-native speakers), employment status (unemployment), and both the individual’s and their parents’ educational background (no or low qualification) to predict literacy skills (Grotlüschen and Buddeberg, 2020). Besides these, a series of further factors have been proposed to explain low literacy skills in adulthood. However, studies examining these components typically focus on only one domain at a time. In the following, we will consider components of three domains (linguistic, domain-general, and numerical) that might be linked to low literacy skills in adulthood.

Performing poorly on various phonological processing tasks is a typical symptom of the neurodevelopmental disorder developmental dyslexia and also characterizes adults with low literacy skills (e.g., Grosche, 2012; Kolinsky and Tossonian, 2022 and see next section). Thus, it was supposed that some of the adults with low literacy skills may suffer from developmental dyslexia which remained undiagnosed or untreated in their childhood (e.g., Bar-Kochva et al., 2021; Greenberg et al., 1997; Vágvölgyi et al., 2021).

Studies assessing the prevalence of developmental dyslexia or learning disabilities among adults with low literacy skills supported this assumption. A study out of the USA revealed that 48% of the participants in the assessed adult basic education programs had a learning disability in childhood, based on self-reports (MacArthur et al., 2010). In another study, 33% of the participants were diagnosed with developmental dyslexia (Gottesman et al., 1996). The LEO 2018 study (Grotlüschen et al., 2019, 2020) showed that the occurrence of developmental dyslexia was more than twice as high among adults with low literacy skills compared to skilled readers (7.0 vs. 2.9%) (Heilmann, 2020).

Consequently, similar to children with developmental dyslexia, adults with low literacy skills may not have properly or completely acquired the stages of reading development (Bulajić et al., 2019; Lachmann, 2018; Lachmann and van Leeuwen, 2014). Based on this long-standing assumption about a possible link between low literacy skills in adulthood and developmental dyslexia (for reviews, see Vágvölgyi et al., 2016, 2021), the *Multi-level Framework of Developmental Disorders* (Lachmann et al., 2022) was taken as a core structure for our measurements.

The framework describes that the cognitive functions required for the acquisition of literacy skills (*information processing level*) may be impaired due to causal neurobiological deficits (*neurobiological level*). The impairments of the cognitive precursor functions would lead to specific learning deficits (i.e., in reading, writing, and arithmetic; *skill level*). It would then lead to reduced performance in school (*academic achievement level*) and to a wide range of further symptoms (e.g., anxiety, *secondary level*). The direction of the transition of deficits can be reciprocal, and individual and environmental factors may also impact the process.

#### 1.1.1 Linguistic domain

The most elementary step in reading acquisition is to learn *phoneme-grapheme correspondences* (Morais et al., 1979). Several studies have revealed that adults with low literacy skills have difficulties with successfully manipulating phonemes when compared to age-matched adults (Grosche, 2012) or to reading-level-matched children (Eme et al., 2014; Greenberg et al., 2002; Greenberg et al., 1997; Grosche, 2012; Kolinsky and Tossonian, 2022; Thompkins and Binder, 2003). In English, phoneme recognition, phoneme deletion, and phonological spelling were found to explain the reading difficulties of adults with low literacy skills (Thompkins and Binder, 2003); in German, phonological awareness, verbal working memory (WM), and rapid automatized naming (RAN) of phonological stimuli (i.e., three areas of phonological processing) were found to explain reading difficulties (Grosche and Grünke, 2011).

The languages used in the mentioned studies are explicitly addressed here because transparent and opaque orthographies might lead to differing results in particular with regard to phonological and orthographic predictors. In more transparent orthographies with higher level of consistency and regularity (e.g., German), children can reach a certain level of reading accuracy much faster than learners of opaque languages (e.g., English; Landerl and Wimmer, 2008; Seymour et al., 2003). Thus, readers of transparent orthographies have an advantage compared to readers of opaque languages during reading acquisition, because they can read all pronounceable sequences of letters without attending the meaning of the written materials. A meta-analysis demonstrated a moderate-to-strong correlation between lexical access (as measured by *RAN*) and four reading skills: word reading, text reading, non-word reading, and reading comprehension. Although the effects were always larger in the case of individuals with developmental dyslexia when compared to skilled readers or unselected samples (i.e., random sampling in participant selection), the reading level did not appear to moderate the connection (Araújo et al., 2015). Regarding adults with low literacy skills, they performed better than reading-level-matched typical reading children in color, number, and letter naming, but worse than literate adults (Grosche, 2012).

The importance of *oral language comprehension* and decoding in reading comprehension is well known since the model of Gough and Tunmer (1986, Simple View of Reading). This role has not only been confirmed in children learning to read, but in adults with low literacy skills (Sabatini et al., 2010; Talwar et al., 2020).

#### 1.1.2 Domain-general factors

Linguistic processes and representations (i.e., phonological-orthographical, lexical, semantic) have, thus far, been the primary focus when studying the profile of adults with low literacy skills. Learning to read, however, requires more than just linguistic processes; other cognitive precursor functions are also essential (see the *Multi-level Framework of Developmental Disorders*; Lachmann et al., 2022).

The visual *attention* span deficit hypothesis claims that developmental reading disorders are not necessarily a consequence of phonological deficits but of attention impairments (Bosse et al., 2007; Lobier et al., 2012; Valdois et al., 2004). In selective attention, adults with low literacy skills performed as poorly as typical developing children aged 6–8 years (Eme, 2006). When adults with low literacy skills were compared with literate adults in mental alertness and divided attention, they performed significantly worse (Van Linden and Cremers, 2008). Sustained attention, the ability to constantly maintain focus on a task or text, is also an important factor during reading (e.g., Stern and Shalev, 2013). However, to our knowledge, sustained attention in adults with low literacy skills has not yet been studied.

*Inhibition* refers to the executive function that keeps one focused and able to ignore distracting stimuli (Diamond, 2006) and was found to be associated with the reading comprehension of children with and without learning impairments (e.g., Altemeier et al., 2008). Some studies also revealed an impairment of inhibitory control in adults with developmental dyslexia (e.g., Smith-Spark et al., 2016). Although an inhibitory impairment seems to be related to reading difficulties, to our knowledge, it has never been assessed in a sample of adults with low literacy skills.

Reading comprehension requires a certain degree of *WM* capacity from the reader (Baddeley, 2003; Daneman and Merikle, 1996). When the *WM* capacity of adults with low literacy skills was assessed, they consistently performed worse in comparison to fourth graders (Eme, 2006), reading-level-matched children (Thompkins and Binder, 2003), and age-matched adults (Grosche, 2012).^1^ Thus, it seems that a WM deficit might contribute to poor text comprehension and low literacy skills.

#### 1.1.3 Numerical domain

Including the numerical domain as an underlying component of poor reading comprehension in adulthood may come as a surprise to some readers. At first sight, it does not seem obvious that numerical capabilities might predict reading comprehension. From a more socio-educational perspective, the first definition of functional illiteracy included poor calculation as a function of literacy, alongside reading and writing skills (Euringer, 2016; Tröster and Schrader, 2016; UNESCO, 1979). Although this view conflicts with the older numerical cognition perspective that assumes the independence of core number (magnitude) sense and language (Dehaene and Cohen, 1995), more recent research has revealed linguistic influences on number processing (Dowker and Nuerk, 2016). Nevertheless, to our knowledge, the assessment of basic numerical skills in adults with low literacy skills has been overlooked by research. We will now provide a brief overview of the connection between numerical skills and reading.

A link between language skills and *arithmetic* problem solving has been explored in translingual studies (Göbel et al., 2014), revealing that children calculate faster in languages without number word inversion (e.g., Italian, where 35 is named “thirty-five”) in comparison to languages with number word inversion (e.g., German, where 35 is named “five and thirty”). Throughout development, children master new strategies to solve arithmetic tasks more easily (Imbo and Vandierendonck, 2007). While solving multiplication and subtraction problems did not cause difficulties in the case of adults with developmental dyslexia (De Smedt and Boets, 2010), children with developmental dyslexia were less efficient with single-digit operations (Boets and De Smedt, 2010). In sum, reading deficits and numerical deficits sometimes co-occur, sometimes influence one another, and are even both part of the standard definition of literacy according to UNESCO (1979). Therefore, the arithmetic skills of adults with low literacy skills are worth assessing.

Due to the huge variety of different numerical representations, processes and/or tasks (e.g., Knops et al., 2017), it is important to focus on major representations of numbers. The hallmark representation of numbers is the number *magnitude representation* (e.g., Dehaene and Cohen, 1995). A number magnitude comparison task allows the observation of magnitude processing processes by evaluating the *numerical distance effect* (Moyer and Landauer, 1967). Deficits in the processing of symbolic and nonsymbolic magnitudes were only found in children with dyscalculia and not in children with developmental dyslexia (without comorbid arithmetic deficits; Landerl et al., 2009). We therefore do not expect a close relationship between magnitude processing and reading performance; however, we do expect a close relationship between place-value processing and reading performance. Elementary place-value processing predicts later arithmetic skills (Moeller et al., 2011), especially for more complex calculation tasks. The two-digit number comparison task allows assessing not only the magnitude representation but also the ease of place-value processing via the *unit-decade compatibility effect* (Huber et al., 2016, for a comprehensive model; Nuerk et al., 2011, for a review; Nuerk et al., 2001). This form of place-value processing is especially linked to linguistic processing in adults (Nuerk et al., 2005) and children (Nuerk et al., 2015; Pixner et al., 2011, for a review). Therefore, we expect a closer relationship between place-value processing and reading comprehension, which may be one underlying reason for the observed links between math and language/reading mentioned above.

Children’s *spatial representation of numbers* is different and less efficient compared to adults. The estimation accuracy of the location of numbers on a number line becomes more precise with increasing age and experience (Siegler and Opfer, 2003). Additionally, language properties influence spatial representation of numbers: Children who speak languages without number word inversion (e.g., Italian) perform better, indicated by smaller estimation errors, in comparison to children speaking languages with number word inversion (e.g., German; Helmreich et al., 2011; Shaki et al., 2009). Regarding children with developmental dyslexia, the comparisons with typically developing children revealed no difficulties in representing numbers on the 0–100 linear number line (Landerl et al., 2009).

Comorbidities between dyslexia and dyscalculia have frequently been reported (e.g., Cheng et al., 2018; Landerl et al., 2009; Landerl et al., 2013; Peters et al., 2020; Wilson et al., 2015). While some studies report common deficits such as visual perception deficits (Cheng et al., 2018), most researchers agree that we are looking at distinct profiles with generally little overlap (e.g., Landerl et al., 2009). Nevertheless, more recent research suggests that people with comorbid dyslexia and dyscalculia might be more severely impaired than individuals with pure dyslexia and dyscalculia alone. For instance, Peters et al. (2020) observed an additive effect for comorbid dyslexia/dyscalculia compared to isolated dyslexia and dyscalculia groups in the context of numerical magnitude processing. Since the problems of children with reading/writing impairments and/or arithmetic impairments cannot be fully described by investigating just one of these areas alone, it seems advisable to assess both reading and arithmetic skills. Thus, we find that adults with reading/writing impairments should also be assessed for arithmetic impairments, and whether and how both are related.

To our knowledge, no studies have examined basic mathematical (i.e., numerical) skills and their potential role (alongside linguistic skills and domain-general functions) concerning low literacy skills in adulthood. Nevertheless, we assume that low literacy skills in adulthood are not restricted to reading and writing skills but might also include basic domain-general and numerical impairments.

### 1.2 Aims

Research and practice concerning low literacy skills in adulthood have most often focused on reading and writing skills. Numerical skills, on the other hand, have been largely ignored in research and typically handled separately in practice. In order to better understand the components behind low literacy skills in adulthood, more than just the linguistic factors should be investigated. Therefore, in this study, we aimed to explore the contribution of linguistic, domain-general, and numerical factors to low literacy skills, which may provide valuable information to instructors of adult basic education classes in developing their course materials.

## 2 Methods

### 2.1 Sample recruitment and participants

This study considers adults with low literacy skills to be those who had attended school for at least 6 years without graduating and struggle with written materials. In Germany, the VHSs (Volkshochschule, adult education center) are the biggest providers of basic education courses (Löffler and Weis, 2016) and offered 1,554 classes in 2021 (Ortmanns et al., 2023). Therefore, seven VHSs’ basic education courses, two basic education centers, and one vocational school for adults in four federal states of Germany were contacted to recruit adults with low literacy skills. The sample also included young adults with high risk of low literacy, recruited from eight vocational schools for young adults.

Among other factors, the positive attitude of the basic education class teachers toward this study motivated the participants to attend the study. Participants often view it as opportunity to try themselves out in a new situation but in a safe environment (i.e., their classroom). In total, 191 participants were tested. The participants gave their informed written consent and received a compensation at the end of the testing. The participants from basic education courses and the adults from vocational training received a payment of 75€ for attending the testing. Because the federal state’s regulation meant that the participants from the vocational schools could not get a direct payment, we instead gave 150€ to their class fund. The study was approved by the Faculty of Economics and Social Sciences Ethics Committee of the University of Tuebingen in 2016.

### 2.2 Materials

Several aspects should be considered in the selection of the materials: (1) The instructions should be easily understandable to avoid floor effects, (2) the tests should be short and reliable, and (3) the tests should fit the proficiency level of adults with low literacy skills. Most of the selected materials were standard tests with reliability and validity measures (see Table 1); however, as there are almost no tests with specific validation in samples of adults with low literacy skills, some of the tasks were custom-made or originally constructed for children (Table 1; Supplementary Table S1).

**Table 1:** Descriptive characteristics and reliability measures of the materials.

Component	Task	Measurement	Norm sample	*N*	*M* (*SD*)	Reliability
Literacy						
Text comprehension	ELFE 1–6	Correct	4th grade	36	15.05 (4.05)	r_S-B_ = 0.72
Linguistic factors						
Word reading	SLRT-II: words (Version A)	Correct	Adults	121	115.17 (19.50)	r_p_ = 0.93
	SLRT-II: words (Version B)			120	120.21 (17.31)	
Pseudoword reading	SLRT-II: pseudowords (Version A)	Correct	Adults	121	73.90 (18.55)	r_p_ = 0.95
	SLRT-II: pseudowords (Version B)			120	75.18 (18.11)	
Text reading	ZLT-II	Errors	7th grade	83*	4.74 (2.99)*	r_tt_ = 0.72
Phonological awareness	BAKO 1–4: subtest 4	Correct	4th grade	227*	54.30 (12.32)*	r_S-B_ = 0.75
	BAKO 1–4: subtest 7					r_S-B_ = 0.82
RAN	ZLT-II: board 1	RT	1st–4th grade	664*	22.86 (5.00) s*	r_tt_ = 0.94
	ZLT-II: board 2				32.27 (9.62) s*	r_tt_ = 0.89
Oral language comprehension	ADST: semantic comprehension	Error rate	*Grund-, Hauptschule* 10th grade	4,350	2 (5)	r_S-B_ = 0.80
	ADST: grammatical comprehension				8 (10)	r_S-B_ = 0.73
Domain-general factors						
Sustained attention	FAIR-2 (Version A)	K-value	9–85 years	1,349	303.69 (102.14)	r_S-B_ = 0.91
	FAIR-2 (Version B)			842	345.30 (95.04)	r_S-B_ = 0.91
Inhibition	TAP: Go/NoGo	False alarm	Adults	439	3.31 (4.84)	r_S-B_ = 0.68
Simple working memory	1-back: letters	Correct	Own sample	154	19.82 (8.24)	r_S-B_ = 0.64
	1-back: shapes	Correct	Own sample	153	20.11 (7.36)	r_S-B_ = 0.59
Complex working memory	2-back: letters	Correct	Own sample	151	15.56 (6.56)	r_S-B_ = 0.82
	2-back: shapes	Correct	Own sample	147	14.52 (5.77)	r_S-B_ = 0.81
Numerical factors						
Arithmetic	Additions	Correct	–	–	–	–
	Multiplications	Correct	–	–	–	–
Magnitude processing and place-value integration	Magnitude comparison task	Mean RT	Own sample	162	1.09 (0.43) s	r_S-B_ = 0.99
Spatial representation of numbers	Linear number line estimation task	Absolute errors	Own sample	154	5.95 (4.05)	r_S-B_ = 0.88
Demographic and control variables						
Non-verbal intelligence	LPS-2 (Version A)	Correct	9th grade	572	20.69 (3.65)	r_S-B_ = 0.77
	LPS-2 (Version B)			682	20.82 (4.28)	r_S-B_ = 0.89

r_tt_ refers to the test–retest-reliability, r_S-B_ refers to the split-half reliability with Spearman-Brown correction, r_p_ refers to the parallel forms. *Data only available for the whole test.

#### 2.2.1 Assessment of the level of literacy

Different measures of reading, such as reading speed, decoding, and comprehension (e.g., Boltzmann and Rüsseler, 2013; Eme et al., 2010), are used to assess literacy skills among adults. We consider reading comprehension to be a core difficulty for adults with low literacy skills (for reviews, see Bar-Kochva et al., 2019; Bulajić et al., 2019; Vágvölgyi et al., 2016, 2021). Therefore, literacy skills were assessed with a speeded text comprehension task, originally designed for children. The test contains 13 short texts and 20 multiple choice questions to be answered within a limited time (ELFE 1–6, Ein Leseverständnistest für Erst- bis Sechstklässler; Lenhard and Schneider, 2006).

#### 2.2.2 Assessment of linguistic factors

Based upon the previous literature (see Introduction), basic reading skills, phonological processing, RAN, and oral language comprehension functions were assessed as possible predictors of text comprehension skills.

##### 2.2.2.1 Skill level

Basic reading skills were assessed at the word and text levels. The participants were first asked to read aloud a list of frequent words and a list of pseudowords within a limited time (SLRT-II, Salzburger Lese- und Rechtschreibtest-II; Moll and Landerl, 2010), and then to read aloud a half-page text (ZLT-II, Zürcher Lesetest-II; Petermann and Daseking, 2012).

##### 2.2.2.2 Information processing level

Phonological awareness was assessed using two tasks. In the first task, the participants heard words and pseudowords and had to manipulate them by changing the first and second phonemes and then saying the new word aloud (subtest Phonemevertauschung). In the second task, they were required to say the words backwards (subtest Wortumkehr, BAKO 1–4, Basiskompetenzen für Lese- Rechtschreibleistungen; Stock et al., 2003).

Lexical access was assessed by a RAN task with drawn objects in two 5×6 matrices (ZLT-II; Petermann and Daseking, 2012). In the first board, five objects (fish, heart, car, glass, and sun) repeated themselves, while in the second, all items were unique (e.g., fork, bone, apple, umbrella, and candle).

Oral language comprehension was also assessed by two tasks. In the first task, participants had to answer multiple choice questions about two stories that they listened to (semantic comprehension). In the second task, single sentences were played and the multiple choice questions aimed at the syntagmatic content (grammatical comprehension; ADST, Allgemeiner Deutscher Sprachtest; Steinert, 2011).

#### 2.2.3 Assessment of domain-general functions on the information processing level

Based upon the previous literature (see Introduction), sustained attention, inhibition (as a subfunction of executive functions), and working memory functions were assessed as possible domain-general (non-linguistic) predictors of text comprehension skills.

Sustained attention was assessed by a selection task. The participants had to select target items from a list within a limited time while following the strict rule of marking (FAIR-2, Frankfurter Aufmerksamkeits-Inventar 2; Moosbrugger et al., 2011).

Inhibition was assessed by a classical Go/NoGo task. Participants had to press the response key when the target item (“x”) appeared and inhibit their urge when the distractor (“+”) appeared (TAP 2.3, Testbatterie zur Aufmerksamkeitsprüfung Version 2.3; Zimmermann and Fimm, 2012).

Working memory was assessed by more rounds of *n*-back tasks. Such a task requires multiple processes such as storage (maintenance), manipulation of the information, updating, and matching (Jaeggi et al., 2010; Owen et al., 2005). Here, the 1- (simple) and 2-back (complex) task with verbal (letters: C, F, L, V) and visual (geometrical shapes: circle, triangle, square, star) stimuli were used. In each round, a sequence of stimuli was presented, and the participants had to indicate whether the item presented *n* trials ago is the same or different from the current item. Each round consisted of 30 trials. The items were presented for 500 ms with an interstimulus interval of 2,500 ms. The session started with one or two practice sessions, depending on the participants’ success. The task was administered in PsychoPy 1.83.04 (Peirce, 2007).

#### 2.2.4 Assessment of numerical factors on the skill level

As stated above, we aimed to explore some basic numerical skills that have been shown to be influenced by language (except for magnitude processing, Dowker and Nuerk, 2016; Fischer and Shaki, 2014; Nuerk et al., 2011; Nuerk et al., 2015, for reviews).

Arithmetic skills were assessed by three speeded blocks of addition and multiplication problems. The participants were randomly assigned to start with either addition or multiplication problems.

Magnitude processing and place-value integration were assessed by a magnitude comparison task (after Nuerk et al., 2001) in which the participants had to decide which number of a two-digit number pair was larger. After a practice session, the testing phase contained 30 compatible and 30 incompatible items with small decade distance, 30 compatible and 30 incompatible items with large decade distance, and 120 within-filler items. One performs better (lower error rate and mean RT) when the distance between the units of the two two-digit numbers is large (e.g., 69_24), called the decade distance effect (magnitude processing, Mann et al., 2011; Nuerk et al., 2004), and when the comparison of both the tens and ones within a two-digit number leads to the same conclusion (both places larger or both places smaller; compatible trial: 24_69, incompatible trial: 29_64), called the compatibility effect (place-value integration, Nuerk et al., 2001). Neither academic training (Wood et al., 2006) nor reading skills (Landerl et al., 2009) have an effect on this. The task was self-paced and administered in PsychoPy 1.83.04 (Peirce, 2007).

The spatial representation of numbers was assessed by the number-line task (Cipora et al., 2015; for a taxonomy of the interval extension association paradigm, see Patro et al., 2014). In the task, the participants must indicate the position of a given number on a 0–100 bounded number line. The task was self-paced and involved 28 one- and two-digit items presented in random order. The presentation was carried out in Java Runtime Environment 8.0 (after Huber et al., 2014).

#### 2.2.5 Assessment of demographic and control variables

Factors such as age, language (mother tongue), schooling (years of formal education), possible impairments/disorders (e.g., vision, hearing, neurological disorders), non-verbal intelligence (IQ approximation score based on subtest 3 of the LPS-2, Leistungsprüfsystem 2; Kreuzpointner et al., 2013), and digit and letter naming skills (letter and number knowledge) might influence literacy skills and thus predict text comprehension skills and were, therefore, included as a control.

### 2.3 Procedure

The tasks were always presented in the same order to avoid unsystematic sources of variance for the regression analysis. Due to the high number of tasks, their complete rotation was not possible due to combinatorial explosion. Alternative methods could not fully rule out order effects; for example, in a Latin Square design, the order remains the same, and only the positions are rotated. Nevertheless, the tasks assessing different factors were mixed to avoid boredom and maintain high motivation. Some of the tasks had parallel versions such as the LPS-2, the SLRT-II, and the FAIR-2. The participants were randomly assigned to the different versions. The 2.5 h testing phase was divided into two sessions (an individual, one-on-one session and a small group session, Supplementary Table S1). The testing was carried out at the participants’ school.

### 2.4 Data preparation

The paper-pencil tasks were scored and the data of the computerized tests trimmed by following the standard procedure (*cf.* Nuerk et al., 2001). Because of the unique testing situation and motivational issues, we had to deal with missing data (Supplementary Table S2). Therefore, with multiple imputations, the data of the whole sample was used to make an estimation of the distribution of the missing datapoints (Donders et al., 2006).

## 3 Results

### 3.1 Sample characteristics

The average age of the participants was 28.31 (*SD* = 17.15; range = 15–68) years. Most of them were men (63%), born in Germany (93%), and stated to be either native or bilingual speakers (69%). More than half of the participants identified their mother (61%) and/or their father (52%) as native speakers of German. On average, the participants spent 10.46 (*SD* = 1.24; range = 6–14) years in school and 54% of them repeated a class at least once in their school career. They often told us further personal details and unpleasant memories from their school time, including impatient and intolerant teachers or everyday confrontation with shame.

The participants were also asked whether they had been diagnosed with any type of learning disabilities; however, because of the high number of unsure and imprecise responses, it had to be omitted from the analysis. An average non-verbal intelligence of 85.03 (*SD* = 25.55) was measured. All participants were able to name at least 9 digits (*M* = 9.99, *SD* = 0.10) and 8 letters (*M* = 9.86, *SD* = 0.43) out of 10 correctly; thus, they were familiar with number and letter symbols (almost all exhibited perfect understanding). Furthermore, they were able to, on average, correctly solve 12.25 (*SD* = 5.75) of 20 short multiple choice text comprehension tasks. Thus, floor and ceiling effects were not observed for the dependent variable (Table 2). Finally, no issues were perceived or reported that might hinder one’s performance during the testing (e.g., visual problems).

**Table 2:** Descriptive information about the sample.

Variable	Mean (SD)
Age (years)	28.31 (17.15)
Schooling (years)	10.46 (1.24)
Non-verbal intelligence (LPS-2, IQ approximation score)	85.03 (25.55)
Digit and letter naming (correct)	9.92 (0.22)
Text comprehension (ELFE 1–6, correct)	12.25 (5.75)

### 3.2 Correlations

Depending on the distribution and the type of the data, different types of correlations were conducted. Thirteen out of the 23 variables correlated with text comprehension skills, including all of the linguistic, four of the demographic and control, two of the domain-general, and one of the numerical variables. According to the strength of the correlations, word/pseudoword reading (i.e., aggregated word and pseudoword reading) showed the strongest linear relationship to text comprehension (*r* = 0.79; Table 3; Supplementary Table S3).

**Table 3:** Results of the correlations ordered according to the relative strength of the relationship to text comprehension.

Domain	Variable	*r*
Linguistic	Word/Pseudoword reading	0.79^***^
Demographic and control	Age	−0.67^***^
Linguistic	Text reading	−0.64^***^
Linguistic	RAN	−0.60^***^
Linguistic	Phonological awareness	0.59^***^
Demographic and control	Non-verbal intelligence	0.58^***^
Domain-general	Sustained attention	0.57^***^
Numerical	Arithmetic: additions	0.56^***^
Linguistic	Oral language comprehension: grammatical comprehension	0.47^***^
Linguistic	Oral language comprehension: semantic comprehension	0.47^***^
Demographic and control	Digit and letter naming	0.37^***^
Demographic and control	Schooling	0.35^***^
Domain-general	Inhibition	−0.32^**^
Numerical	Spatial representation of numbers	−0.26
Domain-general	Simple working memory: 1-back: shapes	−0.25
Numerical	Arithmetic: multiplication	0.24
Numerical	Magnitude processing	−0.23
Domain-general	Simple working memory: 1-back: letters	−0.16
Demographic and control	Mother tongue	−0.14
Domain-general	Complex working memory: 2-back: letters	0.08
Numerical	Place-value integration	−0.02
Demographic and control	Possible impairments/disorders	−0.02
Domain-general	Complex working memory: 2-back: shapes	−0.01

One single measurement per construct was chosen and included in the analysis, thus the two subtests of BAKO 1–4, the SLRT-II and the digit and letter naming were aggregated; *p < 0.05, **p < 0.01, ***p < 0.001.

### 3.3 Predictors of text comprehension

In order to predict text comprehension skills (the core deficit of low literacy skills in adulthood) based on linguistic, domain-general, numerical, and demographic and control variables, a multiple regression was conducted. Low score on the text comprehension task implies lower literacy skills. The predictors jointly accounted for a statistically significant portion of the variance of text comprehension [*F*(23, 167) = 23.27, *p* < 0.001], with an adjusted R^2^ of 0.73. This indicates that the model accounted for 73% of the variability in text comprehension (Table 4) and implies that a major part of the variance in text comprehension can be explained by more basic linguistic and domain-general factors.

**Table 4:** Summary of the multiple regression analysis with text comprehension as continuous dependent variable.

Component	Task	Measurement	β	*b*	*SE*	*t*	*p*	*r*
Linguistic factors								
Word/Pseudoword reading	SLRT-II: words/pseudowords	Correct	0.36	0.07	0.01	4.58	< 0.001	0.79
Text reading	ZLT-II	Errors	−0.07	−0.01	0.01	−1.16	0.28	−0.64
Phonological awareness	BAKO 1–4: subtest 4 and 7	Correct	0.07	0.08	0.06	1.50	0.13	0.59
RAN	ZLT-II: board 1 and 2	*RT*	−0.05	−0.41	0.35	−1.16	0.25	−0.60
Oral language comprehension	ADST: semantic comprehension	Correct	0.12	0.25	0.13	1.98	< 0.05	0.47
	ADST: grammatical comprehension	Correct	0.11	0.28	0.12	2.46	0.02	0.47
Domain-general factors								
Sustained attention	FAIR-2	K-value	0.00	0.00	0.00	0.54	0.59	0.57
Inhibition	TAP: Go/NoGo	False alarm	−0.02	−0.04	0.06	−0.69	0.49	−0.032
Simple working memory	1-back: letters	Error rate	−0.01	0.00	0.01	−0.27	0.79	−0.16
	1-back: shapes	Error rate	−0.09	−0.02	0.01	−2.47	0.01	−0.25
Complex working memory	2-back: letters	Error rate	−0.02	0.00	0.01	10.70	0.09	−0.08
	2-back: shapes	Error rate	0.13	0.00	0.00	1.35	0.10	−0.01
Numerical factors								
Arithmetic	Additions	Correct	0.10	0.03	0.02	1.62	0.11	0.56
	Multiplications	Correct	−0.04	−0.01	0.01	−0.58	0.56	0.24
Magnitude processing	Magnitude comparison task	Decade distance effect	−0.04	0.06	0.27	0.21	0.83	−0.23
Place-value integration		Compatibility effect	−0.03	−0.15	0.26	−0.56	0.57	−0.02
Spatial representation of numbers	Linear number line estimation task	Absolute errors	−0.01	−0.03	0.08	0.44	0.66	−0.26
Demographic and control variables								
		Age	−0.19	−0.05	0.02	−2.48	0.01	−0.67
		Mother tongue	0.02	0.03	0.51	0.06	0.95	−0.14
		Possible impairments/disorders	−0.04	−0.71	0.50	0.06	0.95	−0.02
		Schooling	0.01	0.04	0.21	0.18	0.85	0.35
Non-verbal intelligence	LPS-2: subtest 3	IQ approximation score	0.05	0.01	0.01	1.08	0.28	0.58
Letter and number knowledge	Digit and letter naming	Correct	0.06	1.50	1.16	1.29	0.20	0.37

The number of correct responses given to the ELFE 1–6 indicated the level of literacy skills. The lower the score is, the lower the literacy skills are. One single measurement per construct was chosen and included in the analysis, thus the two subtests of BAKO 1–4, the SLRT-II and the digit and letter naming were aggregated. K-value refers to the continuity score (Kontinuitätswert), i.e., quality x performance. β, standardized beta; b, regression coefficient; SE, standard error of b; r, correlation coefficient.

*R*^2^ = 0.7621, Adjusted *R*^2^ = 0.7294.

Regarding the *linguistic factors* of the joint model, word/pseudoword reading (*b* = 0.07, *p* < 0.001) and oral language comprehension (b_semantic_ = 0.25, *p* < 0.05; b_grammatical_ = 0.28, *p* < 0.05) predicted text comprehension performance, suggesting that weaker word/pseudoword reading skills and weaker oral semantic and grammatical comprehension skills are associated with lower literacy skills. Phonological awareness, number of errors in text reading, and RAN did not predict text comprehension.

Focusing on the *domain-general factors*, error rate in the simple WM task with shapes (*b* = −0.02, *p* < 0.05) predicted text comprehension, indicating that a higher error rate in the simple WM task with shapes is associated with lower literacy skills. Even though it was found to be a predictor, it did not correlate significantly with the dependent variable (*r* = −0.25, *p* = 0.09; Table 3; Supplementary Table S3) and is thus a suppressor variable (Lancaster, 1999). Attention, inhibition, complex WM with letters and shapes, and simple WM with letters did not predict text comprehension.

Regarding the *numerical factors*, the results indicated that numerical skills are independent from literacy skills; none of the variables predicted text comprehension.

Among the *demographic and control variables*, age predicted text comprehension (*b* = −0.05, *p* < 0.05), indicating that higher age in our sample was associated with lower literacy skills. Mother tongue, possible impairments/disorders, schooling, non-verbal intelligence, and digit and letter naming did not predict text comprehension.

## 4 Discussion

The objective of the present study was to explore whether and to what extent basic linguistic, domain-general, and numerical factors explain low literacy skills among adults. The results showed that word/pseudoword reading, oral semantic and grammatical comprehension tasks (linguistic factors), the error rate of the simple WM task with shapes (domain-general factors), and age (demographic and control variable) influence text comprehension performance, whereas numerical factors did not at all predict text comprehension. This implies that a person with low literacy skills might also have low numeracy skills, but these difficulties seem to be either weakly or not significantly linked with each other. This is consistent with earlier suggestions about the comorbidity of dyslexia and dyscalculia (Landerl et al., 2009), which propose different profiles of these learning disorders. In total, the model explained 73% of the variability in the written text comprehension, suggesting that, in our sample, low literacy was largely determined by a combination of age and more basic linguistic and domain-general factors.

When comparing the relative strength of predictors based on their correlations (Tables 3, 4; Supplementary Table S3), it can be seen that word/pseudoword reading, oral language comprehension, and age correlated with and predicted text comprehension. The simple WM task with shapes acted as a suppressor variable when included in the model to enhance explained variance; however, no correlation was found with text comprehension. Our results suggest that low literacy skills in adulthood are mainly predicted by basic linguistic factors, namely word/pseudoword reading and oral language comprehension.

### 4.1 Linguistic factors

Text comprehension was predominantly predicted by linguistic measurements, suggesting that lower literacy skills are associated with other more basic linguistic difficulties, both on the skill and the information processing levels (see *Multi-level framework of Developmental Disorders*; Lachmann et al., 2022). Differences in phonological processing functions between adults with low literacy skills and literate controls on the group level were often found in previous studies (e.g., Eme et al., 2014; Greenberg et al., 1997), in which phoneme recognition, deletion, and phonological spelling explained a significant proportion of the variance in reading skills (55%). Among them, the phonological spelling task including words and pseudowords had the most prominent explanatory power (Thompkins and Binder, 2003). In our study, however, we found only the word/pseudoword reading variable to be a significant predictor of text comprehension skills. The phonological and basic reading measurements were weakly or moderately correlated with one another and moderately or strongly with text comprehension. The word/pseudoword reading variable had the strongest relation to text comprehension. Due to the higher correlation between word/pseudoword reading and the other two tasks, these more purely phonological tasks did not explain any additional variance when word/pseudoword reading was included. Although phonological processes are important for pseudoword reading, they also play a role for other word reading processes (e.g., lexical knowledge). The fact that word/pseudoword reading, a more complex predictor, was the most important suggests that grapheme-phoneme correspondences play a substantial role in determining low literacy, but are not the sole factor.

It was shown that adults with low literacy skills perform worse in listening or oral narrative production tasks in comparison to literate adults (Van Linden and Cremers, 2008); their performance is closer to that of primary school children (Eme, 2006; Eme et al., 2010, 2014). Oral language comprehension is a key component in reading comprehension (Sabatini et al., 2010; Talwar et al., 2020), which was confirmed within our sample by the oral semantic and grammatical comprehension tasks. These linguistic factors remained strong and significant predictors in our study, even though a whole range of domain-general, numerical and control variables were also included in the model.

Although Grosche (2012) provided clear evidence on the poor RAN performance of adults with low literacy skills, our results revealed that lexical access does not provide unique variance in explaining their text comprehension difficulties. Regarding the raw correlations, the RAN performance correlates not only with text comprehension but also with age and the other linguistic predictors (Supplementary Table S3). Among them, word/pseudoword reading and/or oral semantic comprehension could have absorbed the variance of lexical access, which explains low literacy skills in other studies.

### 4.2 Domain-general factors

Although one cognitive variable (simple WM with shapes) predicted text comprehension, there was no raw correlation of these variables. Thus, simple WM with shapes is a suppressor variable in our study; it was not genuinely related to the text comprehension performance, but might have absorbed some irrelevant variance of oral language comprehension. However, this does not preclude that some adults with low literacy skills have low memory capacities; other studies show they do (Supplementary Tables S4, S5; Eme, 2006; Grosche, 2012), but it is not related to their difficulties in understanding written texts.

Despite studies on attention (Bosse et al., 2007; Lobier et al., 2012; Valdois et al., 2004) and inhibition (Potocki et al., 2017) suggesting a clear contribution to developmental dyslexia and reading comprehension, in this study, neither of these functions predicted text comprehension. However, attention and inhibition correlated with most of the linguistic predictors (Supplementary Table S3). This might inflate the effect of these predictors and cancel out the possible effects of attention and inhibition. For instance, the cognitive processes of attention are also needed in some of the linguistic tasks; such tasks cannot be solved without focusing one’s attention on the relevant aspect of the task and inhibiting the irrelevant ones. It might be that the linguistic tasks already included some domain-general components, such as sustained attention or updating, and the domain-general tasks more purely did not explain any incremental variance.

### 4.3 Numerical factors

UNESCO (1979) suggests in their definition that poor numeracy should be part of the definition of functional illiteracy (UNESCO, 1979). The current study indicates that this concept might be appropriate regarding the education policy but seems to be of no relevance for diagnostic purposes. Low literacy skills in adulthood appear to be more closely related to linguistic and domain-general factors, as well as age, than to numerical factors. This supports a numerical cognition perspective that assumes the independence of core number representations and linguistic skills (Dehaene and Cohen, 1995). However, similar to WM, this does not preclude that some of the adults with low literacy skills have difficulties with numerical tasks as well. They do (Supplementary Tables S4, S5), but their poor performance is not related to their deficits in complex text comprehension.

### 4.4 Demographic and control variables

Regarding demographic variables, text comprehension was only predicted by age and indicated that lower literacy skills are associated with higher age. Young adults from vocational schools continuously engage in education and, in theory, practice literacy skills daily at school. In contrast, the older participants of the sample finished their schools several years (or even decades) ago and can avoid the daily use of reading-related materials. This group difference might in fact inflate the effect of age: The observed effects of age may not necessarily be accounting for changes in physical and mental processes due to aging, but instead for confounding variables associated with aging (e.g., schooling, mother tongue, or possible impairments/disorders) in our sample or in society as a whole. A slight association between digit and letter naming and the linguistic predictors (Supplementary Table S3) also appeared, and, similar to the other control variables, could explain this result.

Previous studies have shown that adult basic students have lower than average intelligence (Eme, 2006; Grosche, 2012; Mellard et al., 2011; Rüsseler et al., 2013), which is, consistent with our results, independent of reading skills (Eme, 2006; Grosche, 2012). The average IQ approximation score was a half standard deviation below average, including large individual differences in our sample. However, it was not related to text comprehension: Below-average non-verbal intelligence might not be such a major problem for interpreting our data. However, the non-verbal intelligence correlated with age and the linguistic predictors (Supplementary Table S3), suggesting that it provided enough variation in our sample to explain variance in general. Its correlation with the dependent variable and the linguistic predictors (Supplementary Table S3) suggest that abstract tasks with a speed component seem to be especially difficult for adults with low literacy skills. However, the speed component was already part of some of our linguistic tasks and, therefore, abstract tasks with a speed component may not have explained additional variance in our sample.

## 5 Conclusion and perspectives

The results of our large-scale regression analysis suggest that complex text comprehension (as the hallmark deficit of low literacy skills in adulthood) is mainly predicted by more basic linguistic skills and functions, namely word/pseudoword reading and oral language comprehension. This may suggest that precursor functions, such as phonological and possibly lexical processes, are already impaired in adults with low literacy skills. Moreover, the predicting oral language comprehension variables suggest that adults with low literacy skills do not have an isolated problem with reading. They seem to have more general problems with complex language comprehension (even when texts are presented orally), and these problems uniquely contribute to their performance in text comprehension. The major importance of linguistic factors in our analysis is consistent with previous studies, suggesting the importance of linguistic factors in low literacy (Grosche, 2012; Grosche and Grünke, 2011; Thompkins and Binder, 2003).

What might come as a surprise is that domain-general factors such as WM, attention, and inhibition did not, except for one simple non-verbal WM suppressor variable, explain unique variance in low literacy skills. It is, however, important to note that this does not preclude that cognitive processes play a role (as we found raw correlations between attention and inhibition and text comprehension), but only that they do not add unique incremental variance that is not already captured by the linguistic factors or age. We suggest that, while these variables may be important, they are already implicitly part of other linguistic factors.

Despite the old UNESCO (1979) definition that suggests linguistic and numerical deficits in functional illiteracy, numerical variables did not explain any variance in the model. Therefore, we suggest that functional illiteracy/low literacy and functional innumeracy/low numeracy should be regarded and handled as independent phenomena. Similar to dyslexia and dyscalculia, individuals with low literacy or low numeracy skills appear to have distinct profiles. This does not preclude the possibility that having both low literacy and low numeracy skills can lead to additive impairments. However, while some individuals may experience difficulties in both the linguistic and numerical domains, this does not necessarily indicate a shared underlying symptom.

It is important to note that we did not consider social, cultural, and economic variables in this model, which are believed to be major sources of low literacy skills in adulthood. These factors have been proposed as the primary underlying causes in other studies (Döbert and Hubertus, 2000; Grotlüschen et al., 2019, 2020), which, however, focused exclusively on these social, cultural, and economic variables. Still, our model already predicts 73% of the variance of complex text comprehension. So, even if social, cultural, and economic variables were to explain incremental variance, there is not much variance left to be explained after linguistic and domain-general variables are considered. Thus, if complex text comprehension is considered the core deficit of low literacy skills in adulthood, our results suggest that it can largely be attributed to basic linguistic and domain-general difficulties if these are examined in detail.

As a perspective for intervention, we conclude that training programs should not just consider complex texts but also more underlying skills and precursor functions, in order to improve the participants’ general literacy skills as efficiently as possible. Moreover, although beyond the scope of the present study, it should be noted that further non-cognitive aspects also have to be taken into account for long-term engagement in improving reading via different training programs. For instance, the PISA study (Programme for International Student Assessment), which is measuring different abilities of 15-year-old students, revisited the reading (literacy) framework and expanded the definition with motivational and behavioral aspects (OECD, 2018, 2019a). Finding and maintaining reading interest and enjoyment of reading may affect success in reading (comprehension) already in childhood. Finally, adults with low literacy skills are often faced with feelings of shame, anxiety, or reduced self-esteem in childhood and/or adulthood (Döbert and Nickel, 2000; Nickel, 2007). What is more, the fact that basic linguistic and domain-general factors (word/pseudoword reading and oral language comprehension and simple working memory) contribute to predict written reading comprehension in adults makes these factors a promising target for future interventions. Functional literacy interventions (for a recent meta-analysis, see Kindl and Lenhard, 2023) may also address those basic factors and not only complex text understanding intervention approaches for low literacy may also borrow ideas and even specific programs from a long tradition of reading/writing intervention in dyslexia (see Hall et al., 2023, for a recent extensive meta-analysis), especially according to our results, targeting pseudoword reading and oral comprehension. Such interventions may not only be helpful in adulthood, but it is also conceivable that basic factors like oral comprehension of pseudword reading may also be targeted earlier as a starting point in children, because the endpoint of such basic deficits may be later low literacy. However, to be sure about such statements, we need more longitudinal intervention studies for the low literacy population.

## Data Availability

The raw data supporting the conclusions of this article will be made available by the authors, without undue reservation.

## Footnotes

^1^The applied word span and pseudoword repetition tasks aimed to measure phonological skills, however, are mentioned here because of the WM component.
