# Supplementary material for: Low literacy skills in adults can be largely explained by basic linguistic and domain-general predictors
Source: Front Psychol. 2024 Sep 4;15:1422896. doi: 10.3389/fpsyg.2024.1422896 (PMC11408358; doi:10.3389/fpsyg.2024.1422896)
Supplement: Supplementary file 1 [file Table_1.pdf]

SUPPLEMENTARY MATERIALS

Supplementary Graph 1

Correlation between text comprehension, linguistic, cognitive, numerical, and demographic & control variables.

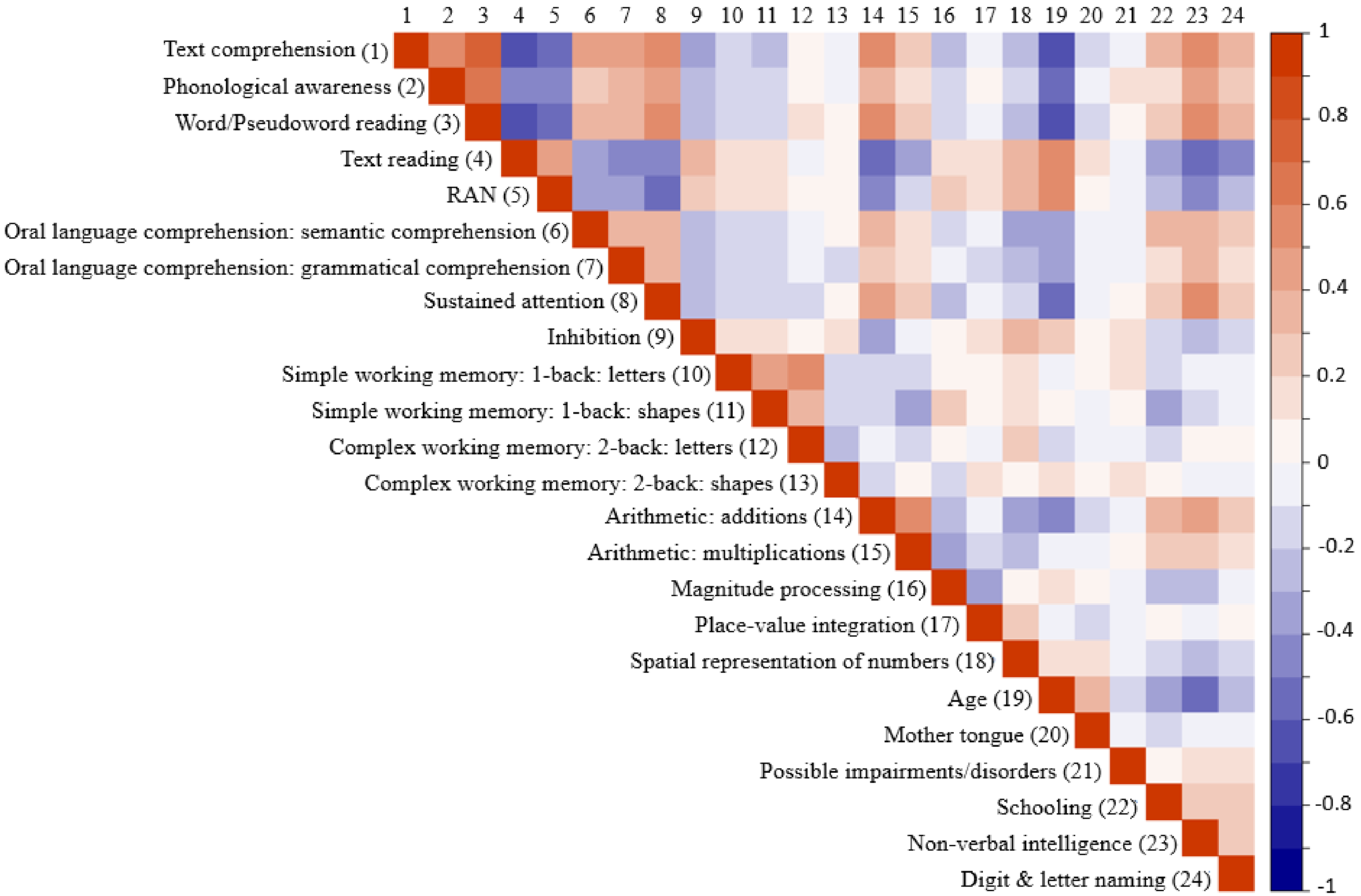

Supplementary Table 1

Testing procedure, details about the materials.

Order	Task	Component	Number of items	Measurements	Standardized/Non-standardized, paper-pencil/computerized/oral presentation
		1st session: Individual testing			
1.	Interview	Demographic information (5)	-	-	Non-standardized, oral
2.	Digit naming task (one- and two-digit numbers)	Digit knowledge (5)	10	Mean of number of correct responses	Non-standardized, oral
	Letter naming task (vowels and consonants)	Letter knowledge (5)	10		
3.	Basiskompetenzen für Lese-Rechtschreibleistungen (BAKO 1-4): subtest 4 (Phonemevertauschung)	Phonological awareness (2)	Max. 14	Mean number of correct responses	Standardized, oral
4.	Leistungsprüfsystem 2 (LPS-2): subtest 3	Non-verbal intelligence (5)	Max. 40 (speeded task)	IQ approximation score	Standardized, paper-pencil
5.	Salzburger Lese- und Rechtschreibtest (SLRT-II): real word reading task	Word reading (2)	Max. 156 (speeded task)	Mean of number of correct responses	Standardized, oral
6.	Salzburger Lese- und Rechtschreibtest (SLRT-II): pseudoword reading task	Pseudoword reading (2)	Max. 156 (speeded task)		Standardized, oral
7.*	Level One-Study: T2, T5, A4, B1 and B4 tasks	Text comprehension (1)	24	-	Non-standardized, paper-pencil
8.	Frankfurter Aufmerksamkeit-Inventar 2 (FAIR-2)	Sustained attention (4)	2	K-value	Standardized, paper-pencil
9.	Basiskompetenzen für Lese-Rechtschreibleistungen (BAKO 1-4): subtest 7 (Wortumkehr)	Phonological awareness (2)	Max. 20	Mean number of correct responses (subtest 4)	Standardized, oral
10.	Ein Leseverständnistest für Erst- bis Sechstklässler (ELFE 1-6): text comprehension for 5th, 6th graders	Text comprehension (1)	Max. 20 (speeded task)	Number of correct responses	Standardized, paper-pencil
11.	Züricher Lesetest-II (ZLT-II): object naming tasks (board 1)	RAN (2)	35	Mean *RT*	Standardized, oral
	Züricher Lesetest-II (ZLT-II): object naming tasks (board 2)		35		
12.	Züricher Lesetest-II (ZLT-II): text reading task (board 6)	Text reading (2)	1	Number of errors	Standardized, oral

(Continued)

Order	Task	Measured construct	Item numbers	Measurements	Standardized/Non-standardized, paper-pencil/computerized/oral presentation
		2nd session: Group testing			
1.*	VERA-8: Deutsch Leseaufgaben: Vorkommnis	Reading comprehension (1)	16	-	Non-standardized, paper-pencil
2.	3 blocks of additions	Arithmetic (3)	Max. 60+60+40 (speeded task)	Mean number of correct responses	Non-standardized, paper-pencil
	3 blocks of multiplications		Max. 60+60+40 (speeded task)		Non-standardized, paper-pencil
3.	Allgemeiner Deutscher Sprachtest (ADST): Teil B - Test 1 (semantic comprehension)	Oral language comprehension (2)	10	Mean number of correct responses	Standardized, paper-pencil
4.	Allgemeiner Deutscher Sprachtest (ADST): Teil B - Test 3 (grammatical comprehension)		10		
5.*	VERA-8: Deutsch Leseaufgaben: Milbe	Reading comprehension (1)	15	-	Non-standardized, paper-pencil
6.	Magnitude comparison task	Magnitude processing (3)	120	Decade distance effect	Non-standardized, computerized[1]
		Place-value integration (3)		Compatibility effect	
7.	1-back task with letters	Working memory (4)	30	Error rate	Non-standardized, computerized[2]
	1-back task with shapes		30	Error rate	
	2-back task with letters		30	Error rate	
	2-back task with shapes		30	Error rate	
8.	Testbatterie zur Aufmerksamkeitsprüfung (TAP 2.3): Go/NoGo	Inhibition (4)	40	Number of false alarms	Non-standardized, computerized
9.	Linear number line estimation task	Spatial representation of numbers (3)	20	Absolute estimation error	Non-standardized, computerized[3]
10.*	Logarithmic number line learning task		20	-	
	Exponential number line learning task		20	-	

Note. The following domains were assessed: (1) literacy, (2) linguistic factors, (3) numerical factors, (4) domain-general factors, (5) demographic & control variables.
* marks the tasks that were not included in the analysis of this study

[1] Presented in PsychoPy 1.83.04 (Peirce, 2007)
[2] Presented in PsychoPy 1.83.04 (Peirce, 2007)
[3] Presented in Java

Supplementary Table 2

Missing data points per task.

Variables	Missing data points (%)
Literacy	
Text comprehension (ELFE 1-6)	0
Linguistic factors	
Phonological awareness (BAKO 1-4, subtest 4)	1.05
Phonological awareness (BAKO 1-4, subtest 7)	1.05
Word reading (SLRT-II)	0
Pseudoword reading (SLRT-II)	0
Text reading (ZLT-II, text reading)	0
RAN (ZLT-II, object naming task 1-2)	0
Oral language comprehension (ADST, semantic comprehension)	13.61
Oral language comprehension (ADST, grammatical comprehension)	13.61
Domain-general factors	
Sustained attention (FAIR-II)	2.09
Inhibition (TAP 2.3., Go/NoGo task)	15.18
Verbal simple working memory (1-back task with letters)	19.37
Visual simple working memory (1-back task with shapes)	19.90
Verbal complex working memory (2-back task with letters)	20.94
Visual complex working memory (2-back task with shapes)	23.04
Numerical factors	
Arithmetic (blocks of additions)	13.61
Arithmetic (blocks of multiplications)	13.61
Magnitude comparison (symbolic comparison task)<br>Place-value integration (symbolic comparison task)	14.66
Spatial representation of numbers (linear number line estimation task)	19.37
Demographic & control variables	
Age	0
Language	0
Schooling	0
Possible impairments/disorders	64.92*
Non-verbal intelligence (LPS-2, subtest 3)	1.57
Digit naming	0
Letter naming	0
**Overall**	**12.74**

Note. * Given the state's regulation, we were not allowed to ask students from vocational schools (n = 124).

Supplementary Table 3

Correlation between text comprehension, linguistic, domain-general, numerical, and demographic & control variables.

Domain	Component	1	2	3	4	5	6	7	8	9	10	11	12
Literacy	Text comprehension (1)	1											
Linguistic factors	Phonological awareness (2)	.59***	1										
	Word/Pseudoword reading (3)	.79***	.61***	1									
	Text reading (4)	-.64***	-.43***	-.67***	1								
	RAN (5)	-.6***	-.48***	-.59***	.48***	1							
	Oral language comprehension: semantic comprehension (6)	.47***	.25	.34***	-.37***	-.39***	1						
	Oral language comprehension: grammatical comprehension (7)	.47***	.35***	.38***	-.4***	-.32**	.37***	1					
Domain-general factors	Sustained attention (8)	.57***	.44	.56***	-.43***	-.51***	.36***	.31**	1				
	Inhibition (9)	-.32**	-.24***	-.24	.32**	.25	-.26*	-.29*	-.29**	1			
	Simple working memory: 1-back: letters (10)	-.16	-.1	-.1	.12	.19	-.19	-.14	-.15	.17	1		

(Continued)

Domain	Component	1	2	3	4	5	6	7	8	9	10	11	12
Domain-general factors	Simple working memory: 1-back: shapes (11)	-.25	-.15	-.14	.16	.18	-.12	-.14	-.15	.1	.44***	1	
	Complex working memory: 2-back: letters (12)	.08	.06	.11	-.03	.03	-.07	-.04	-.11	.04	.54***	.33***	1
	Complex working memory: 2-back: shapes (13)	-.01	-.007	.005	.08	.01	.04	-.16	.05	.13	-.15	-.13	-.23
Numerical factors	Arithmetic: additions (14)	.56***	.34***	.55***	-.53***	-.45***	.31**	.27*	.43***	-.3	-.19	-.19	-.04
	Arithmetic: multiplication (15)	.24	.15	.24	-.33***	-.16	.11	.16	.23	-.05**	-.19	-.31**	-.17
	Magnitude processing (16)	-.23	-.11	-.17	.11	.21	-.13	-.05	-.21	.006	.09	.21	.08
	Place-value integration (17)	-.02	.03	-.06	.17	.19	-.002	-.12	-.05	.12	.03	.01	-.01
	Spatial representation of numbers (18)	-.26	-.18	-.22	.33***	.33***	-.31**	-.23	-.17	.33	.19	.18	.2
Demographic & control variables	Age (19)	-.67***	-.5***	-.68***	.5***	.51***	-.36***	-.31**	-.55***	.2	-.03	.01	-.13
	Mother tongue (20)	-.14	-.004	-.18	.11	.001	-.1	-.02	-.09	.06	.02	-.07	-.02
	Possible impairments/disorders (21)	-.02	.18	.01	-.05	-.07	-.03	-.04	.08	.13	.12	.04	-.03
	Schooling (22)	.35***	.19	.28*	-.33***	-.27*	.3**	.14	.27*	-.19	-.18	-.3**	-.1
	Non-verbal intelligence (23)	.58***	.42***	.51***	-.52***	-.47***	.39***	.33***	.56***	-.27*	-.1	-.19	.02
	Digit & letter naming (24)	.37***	.28*	.33***	-.44***	-.21	.28*	.17	.23	-.18	-.09	-.1	.02

(Continued)

Domain	Component	13	14	15	16	17	18	19	20	21	22	23	24
Domain-general factors	Complex working memory: 2-back: shapes (13)	1											
Numerical factors	Arithmetic: additions (14)	-.12	1										
	Arithmetic: multiplication (15)	.02	.57***	1									
	Magnitude processing (16)	-.14	-.25**	-.3**	1								
	Place-value integration (17)	.15	-.1	-.1	-.32***	1							
	Spatial representation of numbers (18)	.02	-.34***	-.23	.006	.29*	1						
Demographic & control variables	Age (19)	.17	-.42***	-.08	.19	-.07	.16	1					
	Mother tongue (20)	.1	-.2	-.05	.005	-.14	.11	.32*	1				
	Possible impairments/disorders (21)	.12	-.04	.04	-.02	-.1	-.07	-.11	-.1	1			
	Schooling (22)	.04	.33***	.25	-.29*	.08	-.18	-.32*	-.17	.06	1		
	Non-verbal intelligence (23)	-.07	.48***	.26	-.26*	-.05	-.25	-.56	-.09	.12	.25	1	
	Digit & letter naming (24)	-.07	.27*	.12	-.1	.04	-.19	-.3*	-.08	.14	.25	.25	1

* *p*<.05, ** *p*<.01, *** *p*<.001

Supplementary Tables 4-5

Since, as previously mentioned, there are no standards as to which criteria are important to consider when recruiting or pooling a low literate sample, or what kind of diagnostic tools should be used to identify them, another complementary analysis was done. Here, the same reading comprehension task (ELFE 1-6, Lenhard, Schneider, 2006) was taken to assess the level of literacy, but it was used as a categorical variable to divide the participants into more and less skilled readers. Thus, the upper, best performing third of the sample was identified as a more skilled low literate group, while the lower, worse performing third as a less skilled low literate group. We have refrained from using the term functional illiteracy in the manuscript because of the unclear definition and the possible stigmatization, but this group with extremely low literacy, lower than many elementary school children, would be largely considered functional illiterates in other studies.

The less skilled low literate group consisted of 69 participants (48 men). Fifty-five of them came from basic education courses and 14 from other schools, while the more skilled low literate group consisted of 72 participants (41 men). Four came from basic education courses and 68 from other schools. Here, again a multiple regression analysis was conducted in focus with the text comprehension performance, but as a categorical dependent variable.

Supplementary Table 4

Descriptive information about the sample with group comparison (two-sample *t*-test).

Component	Measurement	Less skilled low literate group *M (SD)*	More skilled low literate group *M (SD)*	t (d)
Literacy				
Text comprehension (ELFE 1-6)	Correct	5.72 (3.32)	17.97 (1.39)	-28.31*** (-4.85)
Linguistic factors				
BAKO 1-4: task 4/7	Correct	5.11 (5.89)	11.83 (3.12)	-8.41*** (-1.43)
SLRT-II: word/ pseudoword reading	Correct	36.13 (23.01)	81.56 (17.61)	-13.12*** (-2.22)
ZLT-II: text reading	Errors	30.38 (36.87)	2.81 (2.71)	6.20*** (1.07)
ZLT-II: RAN	*RT*	0.66 (1.05)	-0.54 (0.46)	8.66*** (1.49)
ADST: semantic comprehension	Correct	5.08 (2.35)	7.06 (1.68)	-5.75*** (-0.97)
ADST: grammatical comprehension	Correct	4.87 (2.15)	6.85 (2.07)	-5.57*** (-0.94)
Domain-general factors				
FAIR-2	K-value	154.55 (116.49)	303.36 (99.78)	-8.13*** (-1.37)
TAP: Go/NoGo	False alarm	4.28 (5.48)	1.49 (2.59)	3.84*** (0.66)
1-back: letters	Error rate	41.09 (26.68)	31.42 (25.04)	2.22* (0.37)
1-back: shapes	Error rate	46.28 (28.89)	31.76 (30.59)	2.90** (0.49)
2-back: letters	Error rate	48.07 (26.29)	50.51 (29.19)	-0.52
2-back: shapes	Error rate	28.60 (174.52)	29.35 (63.90)	-0.33
Numerical factors				
Additions	Correct	42.83 (22.27)	68.31 (16.81)	-7.64*** (-1.30)
Multiplications	Correct	31.80 (21.44)	42.65 (23.81)	-2.85** (-0.48)
Magnitude comparison	Decade distance effect (z)	0.36 (1.33)	-0.09 (0.81)	2.44* (0.41)
	Compatibility effect (z)	-0.05 (1.46)	-0.04 (0.69)	-0.03
Linear number line estimation	Absolute errors	6.92 (4.35)	5.39 (2.86)	2.45* (0.42)
Demographic & control variables				
Age (years)		42.80 (17.12)	18.29 (7.04)	11.03*** (1.89)
Schooling (years)		9.97 (1.49)	10.75 (0.92)	-3.71*** (-0.63)
Non-verbal intelligence (LPS-2)	IQ approximation score	68.06 (23.76)	98.43 (24.80)	-6.98*** (-1.25)
Digit & letter naming	Correct	9.83 (0.31)	9.98 (0.10)	-3.94*** (-0.66)

* *p*<.05, ** *p*<.01, *** *p*<.001

Supplementary Table 5

Summary of multiple regression analysis with text comprehension as categorical dependent variable.

Component	Task	Measurement	β	b	*SE*	t	*p*
Linguistic factors							
Phonological awareness	BAKO 1-4: task 4/7	Correct	0.01	0.00	0.01	0.34	.74
Word/pseudoword reading	SLRT-II: word/pseudoword reading	Correct	0.20	0.01	0.00	3.19	.002
Text reading	ZLT-II: text reading	Errors	0.06	0.00	0.00	2.40	.02
RAN	ZLT-II: object naming	*RT*	-0.03	-0.04	0.04	-1.00	.32
Oral language comprehension	ADST: semantic comprehension	Correct	0.04	0.01	0.02	0.74	.46
	ADST: grammatical comprehension	Correct	0.05	0.03	0.01	1.95	.05
Cognitive factors							
Attention	FAIR-2	K-value	0.02	0.00	0.00	0.18	.86
Inhibition	TAP: Go/NoGo	False alarm	-0.01	0.00	0.01	-0.65	.52
Simple working memory	1-back: letters	Error rate	-0.04	0.00	0.00	-0.58	.56
	1-back: shapes	Error rate	-0.04	0.00	0.00	-1.24	.22
Complex working memory	2-back: letters	Error rate	0.02	0.00	0.00	0.65	.52
	2-back: shapes	Error rate	0.07	0.00	0.00	1.67	.10
Numerical factors							
Arithmetic	Additions	Correct	0.06	0.00	0.00	2.12	.04
	Multiplications	Correct	-0.04	0.00	0.00	-0.88	.38
Magnitude processing	Magnitude comparison	Decade distance effect	-0.01	0.02	0.03	0.51	.61
Place-value integration		Compatibility effect	0.00	0.00	0.03	-0.04	.97
Spatial representation of numbers	Linear number line estimation	Absolute errors	-0.01	0.01	0.01	0.93	.36
Demographic & control variables							
		Age	-0.12	-0.01	0.00	-2.92	.01
		Mothertongue	0.03	0.06	0.06	0.88	.38
		Disorders	0.02	-0.06	0.06	-0.89	.38
		Schooling	-0.01	0.00	0.03	-0.09	.93
Non-verbal intelligence	LPS-2: subtest 3	IQ approximation score	0.02	0.00	0.00	0.62	.54
Letter and number knowledge	Digit & letter naming	Correct	0.02	0.16	0.13	1.19	.24

Note. The number of correct responses given to the ELFE 1-6 indicated the level of literacy skills. The lower the score is, the lower the literacy skills are. K-value refers to the continuity score (*Kontinuitätswert*), i.e. quality x performance. β, standardized beta; b, regression coefficient; *SE*, standard error of b.

$R^2 = 0.6833$, Adjusted $R^2 = 0.6211$
